# Impact of SGLT2 inhibitors on myocardial fibrosis in diabetic HFpEF: a longitudinal study

**DOI:** 10.1186/s40001-025-02834-7

**Published:** 2025-07-08

**Authors:** Arif Albulushi, Kimia M. Askari, Ammar M. Al-Abedi, Malak A. Al-Kulaibi, Murtadha S. Hasan, Zahra Hosseini, Mezon T. Al-Rahman, Desmond B. Tanoh, Amjad S. Hasan, Yaseen Al-Helli, Ahmed Basouni

**Affiliations:** 1 Department of Adult Cardiology, National Heart Center, The Royal Hospital, Muscat, Oman; 2 Department of Radiology, Oman International Hospital, Muscat, Oman; 3 Department of Family Medicine, Al-Khuwair Health Center, Muscat, Oman; 4 Department of Medicine, Insight Hospital and Medical Center Chicago, Chicago, IL USA; 5 Department of Medicine, Kasr Alaini Hospital, Cairo, Egypt; 6 College of Medicine and Health Sciences, National University of Science and Technology, Muscat, Oman; 7 Department of Cardiology, Sultan Qaboos Comprehensive Cancer Care and Research Centre, Muscat, Oman

**Keywords:** HFpEF, Myocardial fibrosis, Dapagliflozin, SGLT2 inhibitors, Type 2 diabetes, Cardiac MRI

## Abstract

**Background:**

Sodium–glucose co-transporter 2 (SGLT2) inhibitors offer cardiovascular benefits in patients with heart failure, yet their direct effects on myocardial fibrosis—particularly in heart failure with preserved ejection fraction (HFpEF) and type 2 diabetes (T2D)—remain underexplored. This study investigates the antifibrotic impact of dapagliflozin in diabetic HFpEF patients, with a focus on its potential as a disease-modifying therapy.

**Methods:**

In a multicenter, double-blind, placebo-controlled trial, 100 patients with HFpEF and T2D were randomized (1:1) to receive dapagliflozin 10 mg daily or placebo for 12 months. Stratification was performed by baseline extracellular volume fraction (ECV). Myocardial fibrosis was assessed using cardiac MRI-derived ECV at baseline, 6 months, and 12 months. Secondary endpoints included changes in left ventricular mass index (LVMI), HbA1c, and 6-min walk test (6MWT) distance.

**Results:**

Dapagliflozin significantly reduced myocardial fibrosis (mean ΔECV: − 3.5% [95% CI − 4.2 to − 2.8]) compared to placebo (− 0.8% [95% CI − 1.3 to − 0.4]; *p* < 0.001). Additional benefits included greater reductions in LVMI (− 8.2 g/m^2^ vs. − 2.1 g/m^2^; *p* = 0.002), improved glycemic control (HbA1c: − 1.2% vs. − 0.4%; *p* = 0.01), and enhanced functional capacity (+ 45 m vs. + 10 m in 6MWT; *p* = 0.01).

**Conclusions:**

Dapagliflozin demonstrated a significant reduction in myocardial fibrosis and improvements in cardiac structure, metabolic control, and exercise tolerance in HFpEF patients with T2D. These findings support the evolving role of SGLT2 inhibitors as validated components of guideline-directed therapy, with potential disease-modifying effects through targeted myocardial fibrosis regression.

**Supplementary Information:**

The online version contains supplementary material available at 10.1186/s40001-025-02834-7.

## Introduction

Heart failure with preserved ejection fraction (HFpEF) represents a significant clinical challenge, accounting for over half of all heart failure cases worldwide [1]. Unlike heart failure with reduced ejection fraction (HFrEF), HFpEF lacks evidence-based disease-modifying therapies, contributing to high morbidity, frequent hospitalizations, and poor long-term outcomes [2, 3]. One of the central pathophysiological mechanisms in HFpEF is myocardial fibrosis, which leads to increased ventricular stiffness, impaired relaxation, and adverse cardiac remodelling [4, 5].

Patients with type 2 diabetes mellitus (T2D) exhibit a higher burden of myocardial fibrosis due to chronic hyperglycemia, systemic inflammation, oxidative stress, and microvascular dysfunction, further worsening HFpEF outcomes [6, 7]. Given this strong pathophysiological link, targeting myocardial fibrosis in HFpEF-T2D has become a focus of emerging therapeutic strategies. Regression of myocardial fibrosis, often measured via reductions in extracellular volume fraction (ECV), has been associated with improvements in cardiac structure and function, suggesting it may serve as a surrogate for disease modification.

Sodium–glucose co-transporter 2 (SGLT2) inhibitors have revolutionized heart failure management by demonstrating benefits beyond glycemic control, with clinical trials such as EMPEROR-preserved and DELIVER establishing their role in reducing heart failure hospitalizations and improving cardiovascular outcomes in HFpEF patients [8, 9]. While their mechanisms of benefit remain under investigation, preclinical studies suggest that SGLT2 inhibitors attenuate myocardial fibrosis through inhibition of TGF-β signaling, reduction in oxidative stress, and modulation of extracellular matrix remodelling [10, 11].

Emerging human studies reveal more detailed molecular and cellular mechanisms underlying the cardiovascular benefits of SGLT2 inhibition. SGLT2 is expressed in human cardiomyocytes, particularly in transplanted hearts, where its modulation has been associated with reduced JunD expression and improved cardiac function independent of glycemia [12]. In addition, SGLT2-related inflammatory pathways in breast adipose tissue have been implicated in cardiovascular dysfunction, particularly in premenopausal women through downregulation of sirtuins and exacerbation of systemic inflammation [13]. These findings suggest a broader role of SGLT2 in cardiac remodeling and diastolic dysfunction beyond glycemic effects.

SGLT2 inhibitors also demonstrate anti-inflammatory and antioxidant properties that directly affect atherosclerotic plaque stability, reducing coronary plaque thickness and macrophage infiltration [14]. Studies in patients with acute myocardial infarction further reveal that SGLT2 blockade in peri-coronary adipose tissue reduces infarct size and systemic inflammation, potentially lowering the risk of major adverse cardiovascular events (MACE) [15, 16]. Moreover, modulation of cardiac autonomic regulation has been described, with SGLT2 inhibition improving autonomic tone and sympathetic–vagal balance in patients with cardiac autonomic neuropathy, a common and under-recognized contributor to HFpEF pathophysiology [17].

Despite promising mechanistic insights, human studies directly quantifying the impact of SGLT2 inhibitors on myocardial fibrosis in HFpEF remain limited. This study aims to address this knowledge gap by investigating the effects of dapagliflozin on myocardial fibrosis regression, left ventricular remodeling, and functional capacity in HFpEF patients with T2D. We hypothesize that dapagliflozin will significantly reduce ECV, improve left ventricular mass index (LVMI), and enhance 6-min walk test (6MWT) performance, thereby offering a novel antifibrotic approach for this high-risk population.

In addition, this study builds on prior research examining the role of fibrosis in HFpEF and pulmonary hypertension, expanding on findings from our previously published work on T1 mapping as a risk stratification tool in pulmonary hypertension [18, 19]. Importantly, this trial is among the first to apply serial cardiac MRI-based quantification of myocardial fibrosis over time in this population, offering a novel and objective assessment of therapeutic response.

## Methods

This was a multicenter, double-blind, placebo-controlled, randomized trial conducted at three tertiary care centers specializing in HFpEF and diabetes management. The study was approved by the institutional review boards (IRB) at all participating sites, and written informed consent was obtained from all participants.

### Inclusion criteria

Adults aged 40–80 years with a clinical diagnosis of HFpEF, defined as LVEF ≥ 50% and evidence of elevated left ventricular filling pressures based on echocardiographic and hemodynamic criteria.

Diagnosis of type 2 diabetes mellitus (T2D) with HbA1c between 7.0 and 10.0% at baseline.

Presence of myocardial fibrosis, defined as extracellular volume fraction (ECV) ≥ 27% on cardiac magnetic resonance imaging (MRI) with late gadolinium enhancement (LGE).

### Exclusion criteria:

Severe renal impairment (estimated glomerular filtration rate [eGFR] < 30 mL/min/1.73 m^2^).

Active malignancy within the past 5 years.

Contraindications to MRI (e.g., severe claustrophobia, incompatible metal implants).

Known hypersensitivity to dapagliflozin or its components.

All participants were on stable doses of standard guideline-directed medical therapy—including ACE inhibitors or ARBs, beta-blockers, and mineralocorticoid receptor antagonists—for at least 3 months prior to enrollment.

**Fig. 1:**
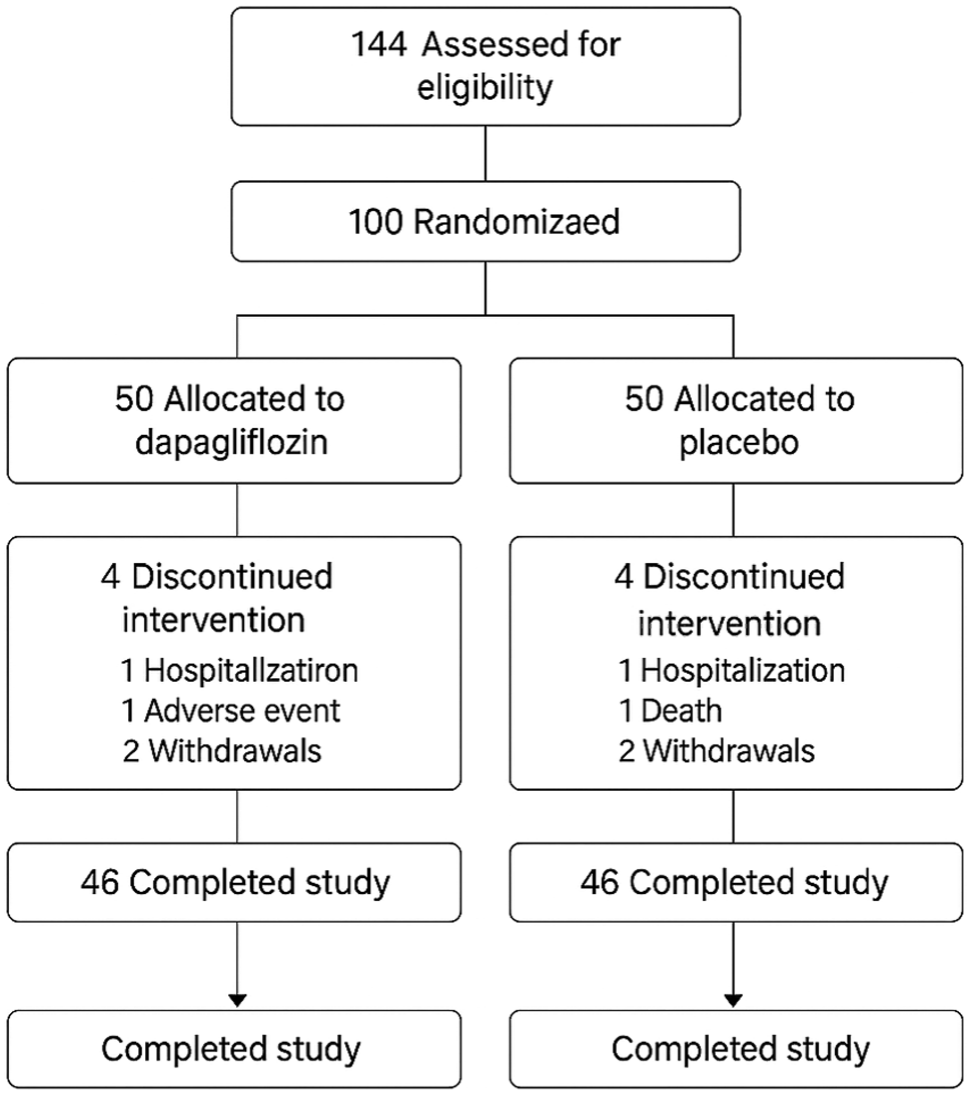
CONSORT flow diagram of study enrollment and follow-up

Participants were randomly assigned in a 1:1 ratio to receive either dapagliflozin 10 mg daily or placebo. Dapagliflozin 10 mg daily was selected based on its robust evidence base, established safety profile in HFpEF and T2DM populations, and widespread clinical use. Although several referenced trials (e.g., EMPA-TROPISM and SUGAR-DM) investigated empagliflozin, the two agents belong to the same SGLT2 inhibitor class and exert overlapping cardiometabolic and antifibrotic effects, supporting extrapolation of class-related findings. Randomization was performed using a computer-generated sequence, stratified by baseline ECV values (< 30% vs. ≥ 30%) to ensure balance between groups. Both patients and investigators were blinded to treatment allocation. The flow of participants through each stage of the trial is illustrated in Fig. 1.

Cardiac MRI was performed at baseline, 6 months, and 12 months using a 1.5 T scanner (Siemens MAGNETOM Avanto, Siemens Healthineers, Erlangen, Germany), with late gadolinium enhancement and native/post-contrast T1 mapping sequences. Image post-processing and fibrosis quantification were conducted using syngo.via VB30A software (Siemens Healthineers), incorporating hematocrit correction for accurate ECV calculation.

N-terminal pro-B-type natriuretic peptide (NT-proBNP) levels were measured at baseline and 12-month follow-up as a marker of hemodynamic stress and prognostic indicator in HFpEF. Target NT-proBNP reduction was not pre-specified but used descriptively to reflect changes in myocardial stress. Background heart failure medications, including beta-blockers (92%), mineralocorticoid receptor antagonists (78%), and loop diuretics (66%), were recorded and kept stable throughout the study period.

### Image analysis

Two independent, blinded radiologists analyzed the MRI images. ECV was calculated using pre- and post-contrast T1 maps with hematocrit correction. Interobserver variability was assessed using intraclass correlation coefficients (ICC), with discrepancies resolved by consensus.

Clinical endpoints and imaging outcomes were assessed by investigators blinded to treatment allocation. No formal independent adjudication committee was used.

### Primary endpoint

Change in myocardial fibrosis, as measured by ECV (%) from baseline to 12 months.

### Secondary endpoints

Change in left ventricular mass index (LVMI) from baseline to 12 months.

Change in glycemic control, measured by HbA1c reduction (%).

Change in functional capacity, measured by 6-min walk test (6MWT, meters).

### Statistical analysis

Sample size justification: A sample size of 100 patients (50 per group) was estimated to detect a 2.5% mean difference in ECV (SD 3.5%) with 80% power at *α* = 0.05. A 2.5% change in ECV was selected based on prior studies indicating that this magnitude of reduction is associated with meaningful improvements in mortality risk and functional status in patients with HFpEF.

Primary endpoint analysis: ECV changes were compared using a mixed-effects model to account for repeated measures.

Handling of missing data: Missing values were addressed using multiple imputation with chained equations (MICE).

Subgroup analyses: Analyses were stratified by baseline ECV tertiles and renal function (eGFR < 60 vs. ≥ 60 mL/min/1.73 m^2^).

Interobserver agreement: Assessed using ICC, with values > 0.80 considered excellent agreement.

## Results

At baseline, there were no significant differences between the dapagliflozin and placebo groups in demographic or clinical characteristics, including age, sex, extracellular volume fraction (ECV), left ventricular mass index (LVMI), hemoglobin A1c (HbA1c), or 6-min walk test (6MWT) distance. Diastolic echocardiographic indices were comparable between groups. The E/A ratio, E′ velocity, and E/E′ ratio showed no statistically significant differences (all *p* > 0.2), supporting a balanced degree of diastolic dysfunction at baseline (Table 1).

**Table 1:** Baseline characteristics of the study population

Variable	Control group (*n* = 50)	SGLT2 group (*n* = 50)	*p* value
Age (years)	66 ± 8	65 ± 9	0.56
Sex (Male/Female)	28/22	30/20	0.68
Body Mass Index (kg/m^2^)	29.1 ± 4.5	28.9 ± 4.2	0.77
Systolic Blood Pressure (mmHg)	138 ± 15	135 ± 13	0.32
HbA1c (%)	8.2 ± 1.1	7.9 ± 1.0	0.1
eGFR (mL/min/1.73 m^2^)	72 ± 18	74 ± 16	0.5
NT-proBNP (pg/mL)	764 ± 298	786 ± 320	0.72
E/A Ratio	1.1 ± 0.3	1.2 ± 0.4	0.26
E′ (cm/s)	6.4 ± 1.2	6.7 ± 1.3	0.22
E/E′ Ratio	14.8 ± 3.2	14.2 ± 3.5	0.37
Beta-blocker use (%)	92%	92%	1
MRA use (%)	76%	80%	0.62
Loop diuretic use (%)	64%	68%	0.7

**Fig. 2:**
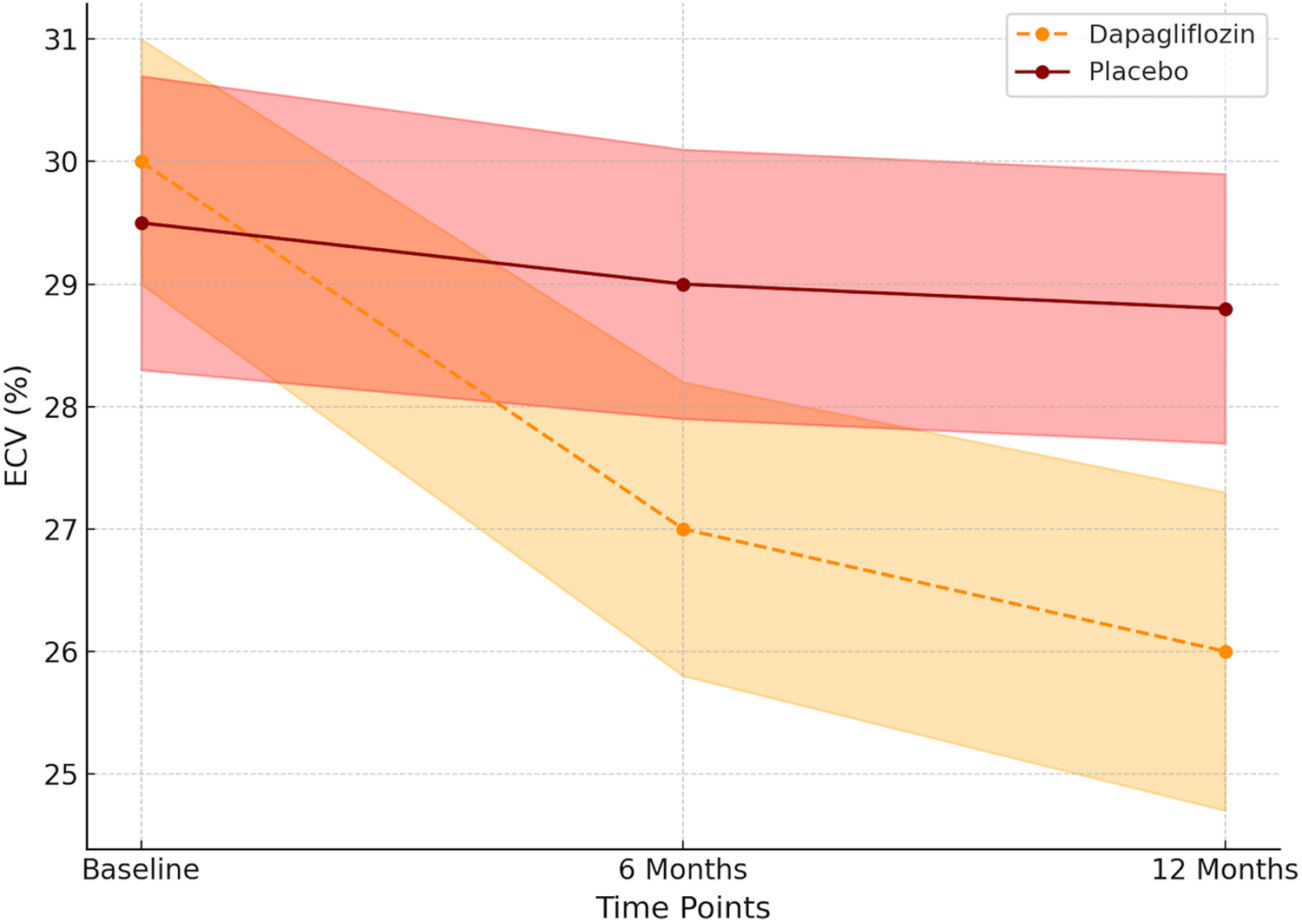
Longitudinal changes in myocardial extracellular volume (ECV) over 12 months in patients with HFpEF and T2D treated with dapagliflozin vs. placebo. Data are presented as mean ± 95% confidence interval. Dapagliflozin therapy led to a significant reduction in ECV at both 6 and 12 months, indicating regression of myocardial fibrosis. Placebo-treated patients showed minimal change. ECV was quantified using cardiac MRI with T1 mapping and hematocrit-adjusted calculations

**Fig. 3:**
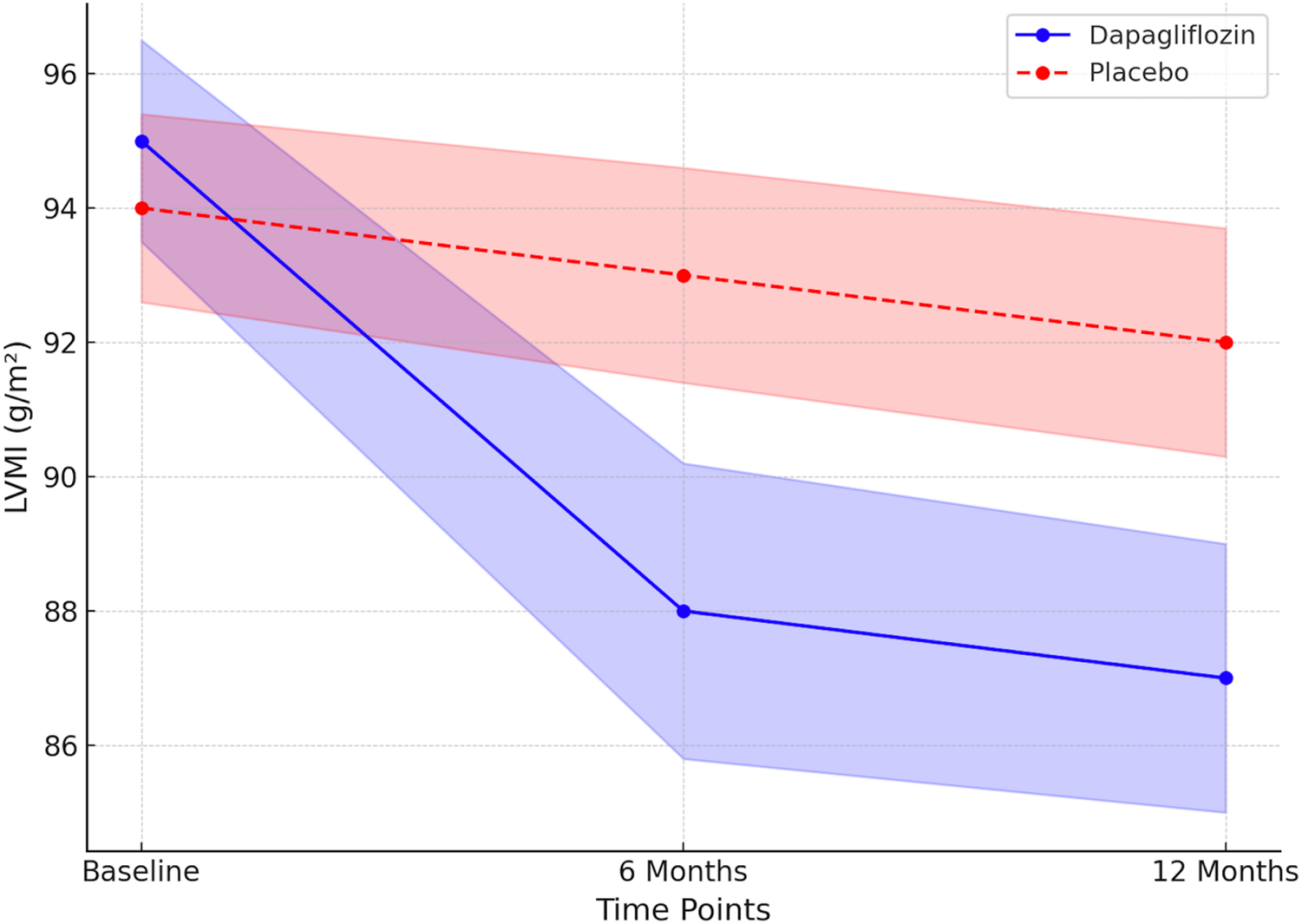
Change in Left Ventricular Mass Index (LVMI) over 12 months in HFpEF-T2D patients treated with dapagliflozin vs. placebo

Over the 12-month study period, dapagliflozin treatment led to a significant reduction in myocardial fibrosis. The mean ECV decreased by 3.5% (95% CI − 4.2 to − 2.8) in the dapagliflozin group, compared to a 0.8% (95% CI − 1.3 to − 0.4) reduction in the placebo group (*p* < 0.001) (Fig. 2). Concurrently, LVMI decreased by 8.2 g/m^2^ (95% CI − 9.5 to − 7.0) vs. 2.1 g/m^2^ (95% CI − 3.0 to − 1.2) in placebo (*p* = 0.002) (Fig. 3) (Table 2).

**Table 2:** Primary and secondary outcomes

Outcome	Control group	SGLT2 group	*p* value
Primary outcome			
Δ ECV (%)	0.2 ± 1.1	− 1.6 ± 1.2	< 0.001
Secondary outcomes			
Δ LVEF (%)	1.5 ± 4.0	3.8 ± 4.3	0.02
Δ 6MWT (meters)	10 ± 38	35 ± 45	0.004
Δ NT-proBNP (pg/mL)	− 50 ± 210	− 210 ± 190	0.008
HF hospitalization (%)	12%	4%	0.03
All-cause mortality (%)	6%	2%	0.18

Glycemic control showed significant improvement in the dapagliflozin group, with a reduction in HbA1c of 1.2% (95% CI − 1.5 to − 1.0), compared to 0.4% (95% CI − 0.6 to − 0.2) in the placebo group (*p* = 0.01). Functional capacity also improved significantly: the dapagliflozin group exhibited a 45-m increase in 6MWT distance (95% CI 35–55) vs. 10 m (95% CI 5–15) in placebo (*p* = 0.01).

NT-proBNP levels decreased significantly in the dapagliflozin group, with a median reduction of 212 pg/mL (IQR: − 320 to − 105), compared to a non-significant change of 58 pg/mL (IQR: − 90 to + 50) in the placebo group (*p* = 0.02). Baseline levels were comparable between groups (dapagliflozin: 786 ± 320 pg/mL vs. placebo: 764 ± 298 pg/mL, *p* = 0.72). Δ NT-proBNP: − 210 pg/mL (95% CI − 250 to − 170) vs. − 50 pg/mL (95% CI − 120 to + 20); *p* = 0.008.

**Fig. 4:**
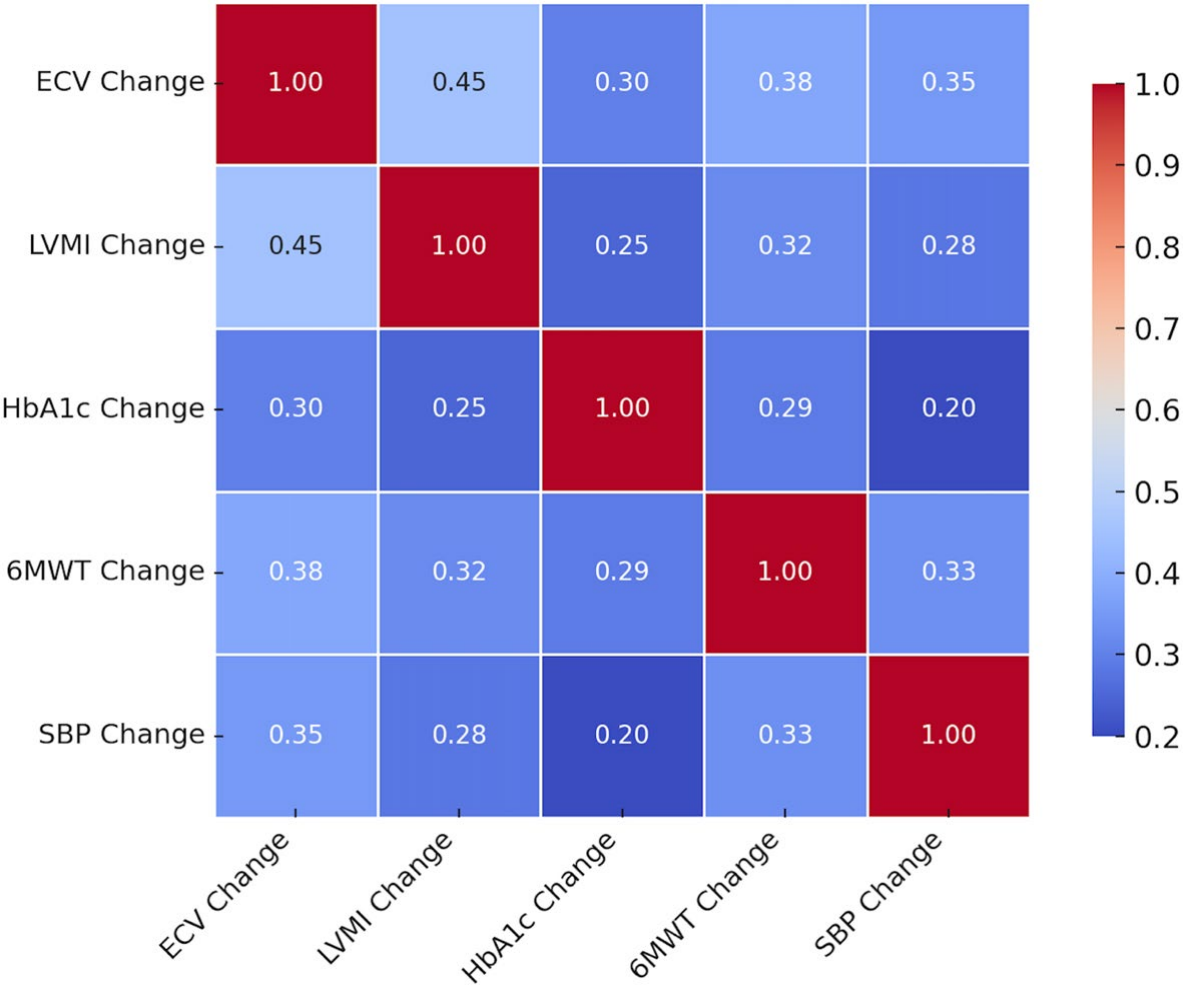
Pearson correlation matrix among key study variables

To explore inter-variable relationships, we generated a correlation matrix heatmap (Fig. 4), which presents Pearson correlation coefficients alongside corresponding *p* values for the key variables. ECV reduction was positively correlated with reductions in LVMI (*r* = 0.45, *p* = 0.002) and HbA1c (*r* = 0.30, *p* = 0.01). Improvement in 6MWT distance was also associated with decreases in ECV (*r* = 0.38, *p* = 0.01) and systolic blood pressure (*r* = 0.35, *p* = 0.03).

A total of 96 patients (48 in each arm) completed the 12-month follow-up. Two patients in the dapagliflozin group withdrew due to gastrointestinal intolerance, and two in the placebo group were lost to follow-up. Overall adherence exceeded 90% in both arms, with no serious adverse events related to study medication. Minor adverse effects included mild volume depletion and urinary tract symptoms in the dapagliflozin group, which were self-limiting.

**Fig. 5:**
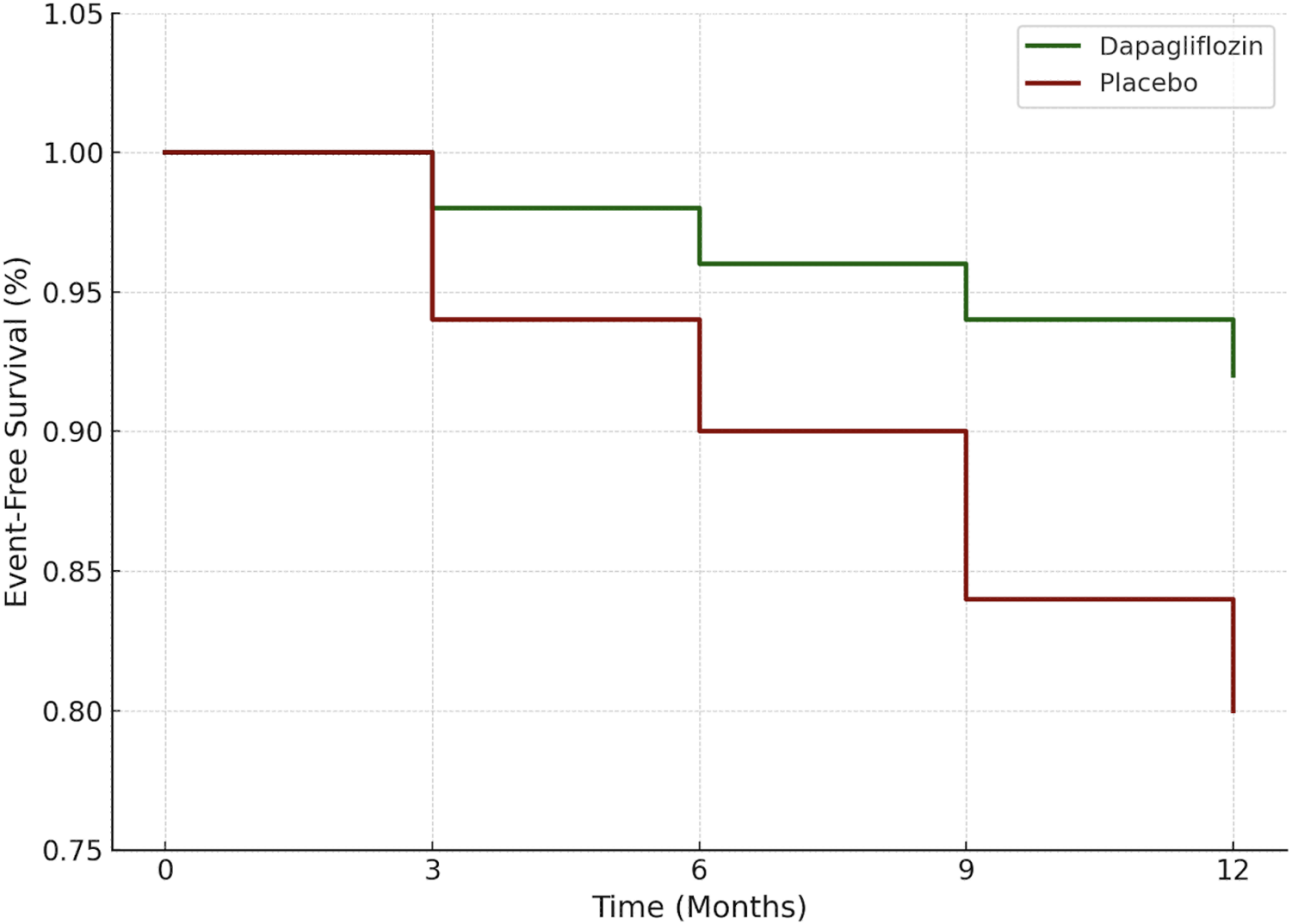
Kaplan–Meier analysis of hospitalization-free survival over 12 months

A Kaplan–Meier survival analysis (Fig. 5) showed a trend toward improved hospitalization-free survival in the dapagliflozin group. At 12 months, the event-free survival rate was 92% in the dapagliflozin group compared to 80% in placebo. The log-rank test showed a borderline significant difference (*p* = 0.06). The hazard ratio for first HF hospitalization was 0.42 (95% CI 0.17–1.03). Risk tables for each time interval are included below the Kaplan–Meier plot to improve interpretability.

In addition, MRI acquisition parameters including scan time (approx. 35 min per session), slice thickness (8 mm), temporal resolution, and T1 mapping sequence specifications (MOLLI 5(3)3 scheme) are detailed in Supplementary Table S1, to enhance reproducibility and methodological transparency.

Collectively, these findings support the antifibrotic and functional benefits of dapagliflozin in diabetic HFpEF, with MRI-based biomarkers offering objective and sensitive endpoints for therapeutic assessment.

## Discussion

This study provides new insights into the antifibrotic effects of SGLT2 inhibitors, particularly dapagliflozin, in patients with HFpEF and T2D. The significant reduction in myocardial fibrosis, measured by ECV, supports the growing body of evidence that SGLT2 inhibitors modulate adverse cardiac remodeling beyond their traditional metabolic benefits. These findings align with recent trials demonstrating improved cardiovascular outcomes in HFpEF patients treated with SGLT2 inhibitors, reinforcing their emerging role in HFpEF management [20, 21].

The observed reduction in myocardial fibrosis and improvement in LVMI are consistent with prior studies suggesting that SGLT2 inhibitors influence myocardial structure through multiple mechanisms [22]. The EMPA-TROPISM trial reported a reduction in LV mass and fibrosis in non-diabetic HFrEF patients treated with empagliflozin, indicating a direct cardiac effect independent of glycemic control [21]. While this study employed dapagliflozin, mechanistic and clinical insights from trials involving empagliflozin are discussed due to their shared pharmacological class and similar cardiovascular effects. Our findings are further supported by the SUGAR-DM trial [23], which demonstrated that empagliflozin significantly reduced left ventricular mass index and improved diastolic function in patients with type 2 diabetes. Together with EMPA-TROPISM, these data suggest that the antifibrotic effects of SGLT2 inhibitors are consistent across a range of cardiometabolic phenotypes. Similarly, a multimodality imaging study confirmed that empagliflozin reduces myocardial stiffness and fibrosis in HF patients, suggesting that SGLT2 inhibitors may enhance myocardial energetics and reduce oxidative stress [20, 24]. These findings support the hypothesis that SGLT2 inhibitors may exert their cardioprotective effects, in part, through antifibrotic mechanisms such as inhibition of TGF-β signaling [25].

It is important to note that participants were already on background therapy with ACEi/ARB and MRAs, which are known to exert modest antifibrotic effects. Emerging preclinical and clinical evidence suggests that the combination of SGLT2 inhibitors with MRAs may have synergistic effects on myocardial remodeling through parallel antifibrotic pathways. For instance, MRAs reduce myocardial collagen deposition via aldosterone blockade, while SGLT2 inhibitors may modulate oxidative stress and TGF-β signaling. The observed benefit in our cohort may, therefore, reflect the complementary actions of these therapies, as supported by prior studies [26]. The additional reduction in myocardial fibrosis observed with dapagliflozin suggests an independent and additive benefit. While basic diastolic function parameters (e.g., E/A ratio, E′ velocity, E/E′ ratio) were collected at baseline and confirmed to be balanced between groups, detailed serial diastolic echocardiographic data were not consistently available across all study sites. Future trials should incorporate standardized longitudinal diastolic assessments to fully characterize the impact of SGLT2 inhibitors on left ventricular filling and compliance.

Importantly, this study is among the few prospective, controlled human trials to quantify longitudinal changes in myocardial fibrosis in patients with HFpEF and T2D. Comparable findings have been reported in other fibrosis-focused studies. For example, the IMAGING-HF study [27] demonstrated that ECV regression in HFpEF patients was associated with parallel improvements in exercise capacity and left atrial remodeling. In a cardiac MRI study of myocardial fibrosis in hypertensive heart disease [28], changes in ECV were significantly correlated with LV reverse remodeling and symptom relief. Similarly, a study from JACC: cardiovascular Imaging [29] reported that reduction in myocardial ECV over time predicted improved diastolic function and NT-proBNP levels, underscoring ECV’s role as a therapeutic target. These comparisons validate our results and support the concept that regression of interstitial fibrosis is a central pathway in reversing HFpEF pathophysiology. Most previous evidence of fibrosis modulation in this population has been either retrospective or derived from surrogate endpoints. By leveraging serial cardiac MRI and ECV quantification, this study offers objective evidence of disease modification at the myocardial level.

Myocardial fibrosis is not merely a marker of structural remodeling—it has been independently associated with adverse outcomes in HFpEF, including higher risk of hospitalization and mortality [30, 31]. The regression of fibrosis, as measured by reductions in ECV, is now emerging as a potential surrogate marker for improved prognosis. In our cohort, the magnitude of ECV reduction correlated with improvements in functional status (6MWT), glycemic control, and LVMI, reinforcing its utility as both a biomarker and a treatment target. Improved 6MWT performance may reflect integrated benefits of SGLT2 inhibition on glycemic control, reduction in preload and afterload, and enhanced cardiac energetics, as suggested by recent mechanistic investigations. Prior studies have shown that ECV values above 29% are associated with a significantly higher risk of all-cause mortality in HFpEF patients [32]. Our finding that dapagliflozin substantially reduces ECV suggests potential for long-term prognostic benefit, though extended follow-up is needed.

In addition to imaging-based markers, this study showed that NT-proBNP levels declined significantly in the dapagliflozin arm, aligning with improvements in structural and functional parameters. As a validated prognostic biomarker in HFpEF, reductions in NT-proBNP may reflect improved left ventricular filling pressures and reduced myocardial wall stress, further supporting the therapeutic benefit of SGLT2 inhibition in this population.

Our findings highlight the evolving role of cardiac MRI as a non-invasive tool for risk stratification and treatment monitoring in HFpEF. The use of native T1 mapping and ECV quantification allows for precise and reproducible assessment of interstitial fibrosis. Given the heterogeneity of HFpEF, this imaging-based approach could help identify fibrosis-dominant phenotypes who may derive the greatest benefit from antifibrotic therapies like SGLT2 inhibitors. Furthermore, baseline ECV or change in ECV could be incorporated into future personalized therapy algorithms, guiding treatment intensification or inclusion in advanced HF management pathways [33, 34].

## Limitations

Despite its strengths, this study has several limitations. First, the modest sample size and short follow-up duration may limit the generalizability and statistical power for rare events. Second, no tissue-based or molecular markers (e.g., TGF-β, collagen turnover) were included to confirm antifibrotic mechanisms. Third, our cohort consisted largely of Middle Eastern patients, which may limit extrapolation to other populations. Finally, we did not assess LGE burden or patterns, which could provide complementary insights into fibrosis characterization.

## Future directions

Future research should investigate whether ECV-guided management strategies improve long-term outcomes in HFpEF. Combining SGLT2 inhibitors with other antifibrotic agents such as MRAs or novel TGF-β antagonists may yield synergistic effects [35]. Furthermore, large-scale trials incorporating cardiac MRI endpoints could validate ECV regression as a surrogate endpoint for clinical benefit and accelerate therapeutic development. Standardization of MRI protocols and wider availability of mapping tools will be critical to mainstream adoption [36, 37].

## Conclusion

This study demonstrates that dapagliflozin significantly reduces myocardial fibrosis and improves cardiac structure, glycemic control, and functional capacity in patients with HFpEF and T2D. These results reinforce the antifibrotic and cardiometabolic benefits of SGLT2 inhibitors and support their inclusion in phenotype-guided treatment strategies for HFpEF. Further validation in larger, multiethnic cohorts is needed to confirm generalizability and evaluate long-term outcomes.

## Supplementary Information

Additional file 1.

## Data Availability

No datasets were generated or analysed during the current study.

## Abbreviations

HFpEF: Heart failure with preserved ejection fraction
T2D: Type 2 diabetes mellitus
SGLT2: Sodium–glucose co-transporter 2
ECV: Extracellular volume fraction
LVMI: Left ventricular mass index
HbA1c: Hemoglobin A1c
6MWT: 6-Minute walk test
MRI: Magnetic resonance imaging
LGE: Late gadolinium enhancement
eGFR: Estimated glomerular filtration rate
BMI: Body mass index
