# Supplementary material for: Impact of SGLT2 inhibitors on myocardial fibrosis in diabetic HFpEF: a longitudinal study
Source: Eur J Med Res. 2025 Jul 8;30:592. doi: 10.1186/s40001-025-02834-7 (PMC12235812; doi:10.1186/s40001-025-02834-7)
Supplement: Supplementary file 1 — Additional file 1. [file 40001_2025_2834_MOESM1_ESM.docx]

**Supplementary Table S1. Cardiac MRI acquisition parameters**

| Parameter | Value |
| --- | --- |
| Scan time | Approximately 35 min per session |
| Slice thickness | 8 mm |
| Temporal resolution | 40–50 ms |
| T1 mapping sequence | MOLLI 5(3)3 scheme |
| Scanner model | Siemens MAGNETOM Avanto 1.5T |
| Imaging software | syngo.via VB30A (Siemens Healthineers) |
| Gadolinium dose | 0.1 mmol/kg body weight |
| Post-contrast delay time | 15 min |
